# Antibacterial, Antibiofilm, and Efflux Pump Inhibitory Properties of the Crude Extract and Fractions from *Acacia macrostachya* Stem Bark

**DOI:** 10.1155/2021/5381993

**Published:** 2021-10-21

**Authors:** Akua Frema Barfour, Abraham Yeboah Mensah, Evelyn Asante-Kwatia, Cynthia Amaning Danquah, Daniel Anokwah, Silas Adjei, Michael Kwesi Baah, Merlin L. K. Mensah

**Affiliations:** ^1^Department of Pharmacognosy, Faculty of Pharmacy and Pharmaceutical Sciences, College of Health Sciences, Kwame Nkrumah University of Science and Technology, Kumasi, Ghana; ^2^Department of Pharmacology, Faculty of Pharmacy and Pharmaceutical Sciences, College of Health Sciences, Kwame Nkrumah University of Science and Technology, Kumasi, Ghana; ^3^School of Pharmacy and Pharmaceutical Sciences, University of Cape Coast, Cape Coast, Ghana; ^4^Department of Herbal Medicine, Faculty of Pharmacy and Pharmaceutical Sciences, College of Health Sciences, Kwame Nkrumah University of Science and Technology, Kumasi, Ghana

## Abstract

Microbial infections remain a public health problem due to the upsurge of bacterial resistance. In this study, the antibacterial, antibiofilm, and efflux pump inhibitory activities of the stem bark of *Acacia macrostachya*, an indigenous African medicinal plant, were investigated. In traditional medicine, the plant is used in the treatment of microbial infections and inflammatory conditions. A crude methanol extract obtained by Soxhlet extraction was partitioned by column chromatography to obtain the petroleum ether, ethyl acetate, and methanol fractions. Antibacterial, efflux pump inhibition and antibiofilm formation activities were assessed by the high-throughput spot culture growth inhibition (HT-SPOTi), ethidium bromide accumulation, and the crystal violet retention assay, respectively. The minimum inhibitory concentrations (MICs) of the crude extract and major fractions ranged from 250 to ≥500 *μ*g/mL. At a concentration of 3.9–250 *μ*g/mL, all extracts demonstrated >80% inhibition of biofilm formation in *S. aureus*. In *P. aeruginosa*, the EtOAc fraction showed the highest antibiofilm activity (59–69%) while the pet-ether fraction was most active against *E. coli* biofilms (45–67%). Among the test samples, the crude extract, methanol, and ethyl acetate fractions showed remarkable efflux pump inhibition in *S. aureus*, *E. coli*, and *P. aeruginosa.* At ½ MIC, the methanol fraction demonstrated significant accumulation of EtBr in *E. coli* having superior efflux inhibition over the standard EPIs: chlorpromazine and verapamil. Tannins, flavonoids, triterpenoids, phytosterols, coumarins, and saponins were identified in preliminary phytochemical studies. Stigmasterol was identified in the EtOAc fraction. This study justifies the use of *A. macrostachya* in the treatment of infections in traditional medicine and highlights its potential as a source of bioactive compounds that could possibly interact with some resistance mechanisms in bacteria to combat antimicrobial resistance.

## 1. Introduction

Antimicrobial resistance (AMR) is a global menace that has jeopardised the effective prevention and treatment of infectious diseases [1]. In bacteria, high levels of drug resistance have been exhibited mainly through the expulsion of antibiotics by membrane-based polypeptides called efflux pumps [2] as well as the development of biofilms that protect them from antimicrobial agents [3]. In the search for agents to combat AMR, natural products from medicinal plants have inspired the discovery of novel drug compounds which may contribute significantly to human health in the future [4]. Previous studies report that plant extracts and secondary metabolites have the potential to counteract certain resistance mechanisms in bacteria to successfully restore the antibacterial effect of hitherto ineffective antibiotics [5, 6]. Given this, much attention has been given to medicinal plants towards the development of new therapeutic agents to combat bacteria pathogenicity and resistance.

*Acacia macrostachya* (Rchb. ex DC) of the family Leguminoseae is one of such useful plants in African traditional medicine. *A. macrostachya* is a prickly shrub growing to about 5 m high with pubescent twigs, armed with rose-like recurved thorns. The plant is widely distributed in savanna areas in West Africa [7]. Traditionally, the roots, stem bark, and leaves are used for the treatment of oral infections including dental caries and gingivitis [7, 8]. The stem bark is used as an analgesic [7] and for treating gastrointestinal ailments such as diarrhoea, dysentery, cholera, and vomiting [7–9]. The root bark is used as an anthelmintic [7, 8]. The leaves are used as an aphrodisiac [8, 9], against sexually transmitted infections such as gonorrhoea and syphilis [7–9] and as an antidote for snakebite poisoning [7, 8]. Previous biological activity studies have confirmed the anticancer [10], antidiabetic [11], antioxidant [12], anti-inflammatory, analgesic [13], antiplasmodial, [14] and antibacterial [15] activities of different parts of *A. macrostachya*. Other works report the presence of oleanane triterpene saponins in the roots as well as phenolic acids and flavonoids with antioxidant activity in the leafy stems [15, 16]. In a continuing effort to investigate the bioactivities of tropical medicinal plants [17, 18], the antibacterial, antibiofilm, and efflux pump inhibition activities of the stem of *A. macrostachya* (Leguminoseae) were studied.

## 2. Materials and Methods

### 2.1. Chemicals

Chemicals used were purchased from Sigma and Aldrich chemicals, UK. Ciprofloxacin and fluconazole were from Denk Pharma GmbH & Co., Germany. Organic solvents were of analytical grade from BDH laboratory supplies, England.

### 2.2. Plant Collection

The stem bark of *A. macrostachya* was collected from Kwahu Asakraka in the Eastern region of Ghana in November 2019 (0°44.1048′N, 6°33.912′W). The plant sample was identified and authenticated by Dr. G. H. Sam, a botanist at the Department of Herbal Medicine, Faculty of Pharmacy and Pharmaceutical Sciences (FPPS), Kwame Nkrumah University of Science and Technology (KNUST). A voucher specimen with reference number KNUST/HM1/2019/S026 was deposited at the Herbarium of the Faculty of Pharmacy and Pharmaceutical Sciences.

### 2.3. Processing and Extraction of Plant Material

The plant material was washed under running water, cut into smaller chunks, sun-dried for 5 days, and coarsely milled. About 2 kg of the powdered stem was Soxhlet extracted using 6 L of methanol (MeOH) at 60°C until the material was completely exhausted (lasted for approximately 8 h). The extract obtained was concentrated under reduced pressure by means of a rotary evaporator. The concentrate was oven-dried at 40°C to yield a dark brown extract weighing 217 g (yield = 10.37% ^w^_w_) subsequently referred to as the “crude extract” or “ACF” in this report.

The crude extract (100 g) was adsorbed onto silica gel 60 (70–230) and partitioned into three main fractions by successive elution with petroleum ether (pet-ether, 300 mL x 3), ethyl acetate (EtOAc, 400 mL x 3), and methanol (MeOH, 400 mL × 3) by column chromatography. This afforded the pet-ether (APF; 2.49% ^w^_w_), EtOAc (AEF; 16.81% ^w^_w_), and MeOH (AMF; 47.59% ^w^_w_) fractions. The crude extract and organic fractions were kept in a desiccator until ready for use.

### 2.4. Phytochemical Screening

Preliminary phytochemical screening to determine the major classes of secondary metabolites in the powdered stem bark was carried out following simple qualitative phytochemical screening methods previously described [19].

#### 2.4.1. Total Phenol Content (TPC)

The total phenolic content of the leaf and stem bark extracts were determined by the Folin–Ciocalteu method [20]. Gallic acid (3.125–100 *μ*g/mL) was used as the reference substance. TPC was expressed as gallic acid equivalent (GAE) in mg/g of dried extract.

#### 2.4.2. Total Flavonoid Content (TFC)

The total flavonoids in the leaf and stem bark extracts were quantified by the aluminum chloride colorimetry method [21]. Quercetin (3.125–100 *μ*g/mL) was used as positive control. A standard curve was prepared using quercetin and the TFC of the extracts extrapolated from this curve. TFC was expressed as quercetin equivalent (QCE) in mg/g of dried extract.

#### 2.4.3. Fractionation of AEF by Chromatography

The ethyl acetate fraction (AEF, 18.3 g) was further fractionated by column chromatography (CC) using silica gel 60 (70−230 mesh). Thin-layer chromatography (TLC) was performed using precoated silica gel 60 TLC plates (GF254 0.25 mm, Alpha Laboratories, UK). Mixtures of pet-ether, EtOAc, and MeOH were used for elution in a gradient manner. Pure isolate was identified by comparing ^1^H, ^13^C NMR, and mass spectral data with published data. Details of the isolation procedure are presented in Supplementary Material (available here).

### 2.5. Antimicrobial Assay

#### 2.5.1. Bacterial Strains and Inoculum Preparation

The microorganisms used for this study included two Gram-positive bacteria *Staphylococcus* aureus (ATCC 25923) and *Streptococcus pyogenes* (clinical strain) and two Gram-negative bacteria *Escherichia coli* (ATCC 25922) and *Pseudomonas aeruginosa* (ATCC 27853). These were provided by the Cell Culture Laboratory of the Department of Pharmacology, KNUST. The microorganisms were cultured on nutrient agar as agar slants in falcon tubes. Standardized cultures were prepared by subculturing in sterile nutrient broth and incubating at 37°C for 24 h. Working cultures were prepared by serial dilution with normal saline to achieve an initial cell count of approximately 1 × 10^5^ CFU/mL.

#### 2.5.2. Evaluation of Antibacterial Activity of the Crude Extract and Fractions

The antibacterial activity of the crude extract and its organic fractions were determined using the High Throughput Spot Culture Growth Inhibition Assay (HT-SPOTi) as described by Danquah et al. and the method description partly reproduced their wording [22]. Briefly, a twofold serial dilution of a stock solution of the test extracts and fractions (1000 *μ*g/mL in 2% dimethyl sulphoxide, DMSO) was done in a PCR half-skirted plate to give a concentration range of 7.8–500 *μ*g/mL. Aliquots of each dilution (100 *μ*L) were dispensed into corresponding wells of a 96-well plate and mixed with 200 *μ*L of molten agar (stabilized at 45°C). The plates were swirled to mix, allowed to set, and then spotted with 2 *μ*L of standardized microbial suspension of the test organism. The plates were allowed to stand for 20 minutes, sealed, and incubated at 37°C for 24 h. Ciprofloxacin was included as the positive control and 2% DMSO, negative control. The presence or absence of growth was determined by visual examination comparing with the control wells.

#### 2.5.3. Biofilm Inhibition Assay

The effect of the crude extract and fractions on biofilm formation by *S. aureus*, *E. coli*, and *P. aeruginosa* was investigated using the microplate crystal violet stain retention method previously described by Abidi et al. and the method description partly reproduces their wording [23]. Overnight cultures of *S. aureus*, *E. coli*, and *P. aeruginosa* prepared in tryptone soy broth (TSB, 1 : 100) were used for the assay. The test samples were solubilized in DMSO (2%) and reconstituted in TSB to achieve a concentration range of 3.9–250 *μ*g/mL. Aliquots of the microbial culture (180 *μ*L) and the test samples (20 *μ*L) were pipetted into a flat bottom 96-well microtiter plate and incubated at 37°C for 24 hours. Negative controls were included in the plate. The experiment was performed in triplicate. After incubation, the supernatant was aspirated and each well was washed with phosphate buffer saline (PBS, pH 7.2) to remove planktonic cells. The adherent biofilms were fixed by drying at 50°C for 30 minutes and stained with 200 *μ*L of 0.1% ^w^/_v_ aqueous solution of crystal violet (CV) for 10 minutes at room temperature. The wells were carefully washed with sterile water and the stain bound to cells solubilized with 200 *μ*L of 95% ethanol. Absorbance was measured at 600 nm using an automated microplate reader (BioTek Synergy H1 Hybrid Multi-Mode Reader: 271230). The mean absorbance was determined, and the percentage inhibition of biofilm was calculated as

(1) $$\begin{array}{c} \text{percentage biofilm in inhibition}=\frac{\text{Abs}\left(\text{control}\right)-\text{Abs}\left(\text{test}\right)}{\text{Abs}\left(\text{control}\right)}\times 100. \end{array}$$

#### 2.5.4. Efflux Pump Inhibition Assay

Efflux pump inhibitory activity was investigated by the ethidium bromide (EtBr) accumulation assay described by Prasch et al. and the method description partly reproduces their wording [24]. Briefly, pure cultures of *S. aureus*, *E. coli*, and *P. aeruginosa* were prepared in Mueller Hinton broth (MHB) and incubated at 37°C with shaking at 150 rpm until an optical density (OD_600_) of 0.8–1 was achieved. The OD of the cultures was adjusted to 0.4 in 10 mL MHB and centrifuged at 3000 rpm for 15 minutes. Pellets were washed and vortexed with phosphate buffer saline (PBS). Aliquots (500 *μ*L) of the bacteria suspension and PBS (500 *μ*L) were pipetted into separate Eppendorf tubes to serve as the test and blank respectively. Filter sterilized glucose solution (0.4%) and different volumes of the extracts and fractions prepared at ½ MIC were added to the test and blank Eppendorf tubes. Aliquots of 100 *μ*L from each mixture were pipetted into a 96-well microtiter plate to which 5 *μ*L of EtBr (0.5 mg/L) was added. Florescence was measured at an emission wavelength of 585 nm and excitation wavelength of 530 nm every 5 minutes over a period of 60 minutes at 37°C using a microplate reader (BioTek, Synergy H1, Vermont, US). Verapamil and chlorpromazine served as reference drugs and DMSO as the negative control.

### 2.6. Data Management and Analysis

All experimental results were analysed using GraphPad prism (version 8 for windows, San Diego, USA).

## 3. Results

### 3.1. Phytochemical Investigation of the Stem Bark

Phytochemical analysis on the dried powdered stem bark of *A. macrostachya* revealed the presence of various classes of phytochemicals including tannins, saponins, triterpenoids, phytosterols, flavonoids, and coumarins. Alkaloids were not detected (Table 1).

Quantitative determination of the total phenolic content (TPC) and total flavonoid content (TFC) for the stem bark was also determined. TPC was obtained from a standard curve of gallic acid using the equation *y* = 0.01422*x* + 0.1326; (*R*^2^ = 0.9778) and was expressed as gallic acid equivalent (GAE). TFC was determined from a standard curve of quercetin using equation *y* = 0.007687*x* + 0.1365; (*R*^2^ = 0.9106) and was expressed as quercetin equivalent (QCE) (calibration curves for TPC and TFC are provided in attached supplementary material). Both phenolic and flavonoid contents varied with respect to the solvent used for extraction with methanol extracting the highest amounts of phenolic and flavonoid constituents. The results are summarized in Table 2.

Fractionation of the bioactive EtOAc fraction by chromatography resulted in the isolation of a known phytosterol, stigmasterol [25], identified based on its ^1^H and ^13^C NMR data (see supplementary material).

### 3.2. Antibacterial Activity of *A. macrostachya* Stem Bark Extract and Major Fractions

The crude extract and its fractions showed varying degrees of antimicrobial activity against Gram-positive and Gram-negative bacteria in the HT-SPOTi test. The MICs for susceptible bacteria ranged from 250 to ≥500 *μ*g/mL (Table 3). The crude extract showed the highest inhibition towards the test organisms with *E. coli* being most susceptible at MIC of 250 *μ*g/mL. The pet-ether fraction showed no inhibition against all test organisms. None of the test samples inhibited the growth of *S. aureus*. Ciprofloxacin showed varying inhibitory activities on the microorganisms with the highest effect against *S. pyrogens* (MIC- 0.3125 *μ*g/mL). *S. aureus* was resistant to ciprofloxacin at the highest concentration tested (10 *μ*g/mL).

### 3.3. Biofilm Formation Inhibitory Effect of *A. macrostachya* Stem Extract and Fractions

**Figure 1:**
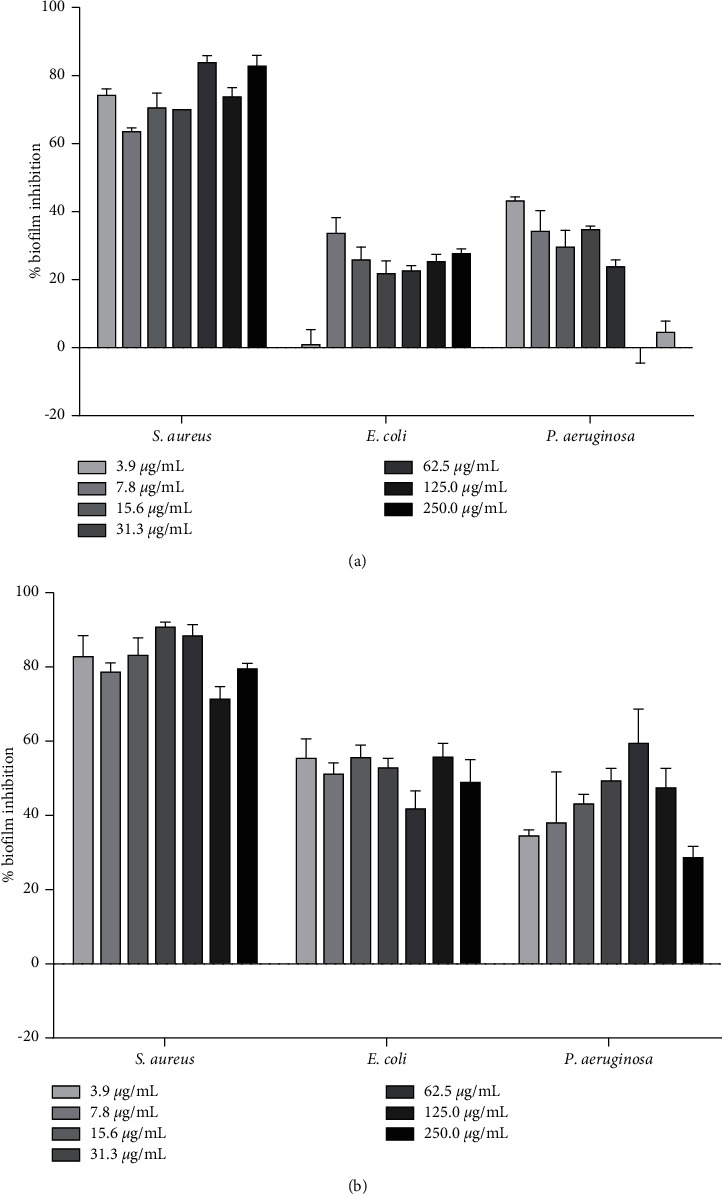
Antibiofilm formation activity of the crude extract, ACF (a), and the MeOH fraction, AMF (b), in *S. aureus*, *E. coli*, and *P. aeruginosa*.

**Figure 2:**
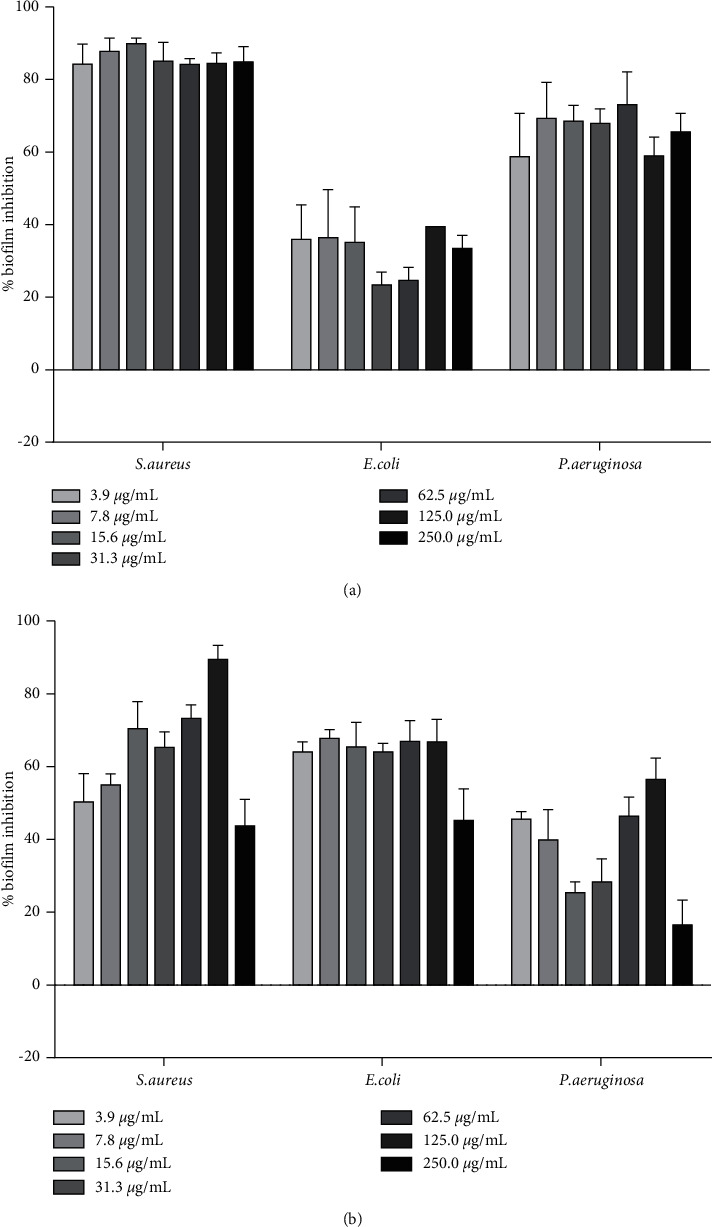
Antibiofilm formation activity of the EtOAc fraction, AEF (a), and the pet-ether fraction, APF (b), in *S. aureus, E. coli*, and *P. aeruginosa*.

The crude extract (ACF) and major fractions (AMF, AEF, APF) were tested for biofilm formation inhibitory effect against *S. aureus*, *E. coli*, and *P. aeruginosa* at a concentration range of 3.9–250 *μ*g/mL. The percentage biofilm inhibition for all samples is demonstrated in Figures 1 and 2. For all test extracts, the antibiofilm effect was not concentration-dependent. In *S. aureus*, all samples demonstrated remarkable inhibition with the highest effect given by the ethyl acetate fraction (AEF). The percentages of biofilm inhibitions recorded against *S. aureus* were AEF (84–90%) > AMF (71–90%) > ACF (63–83%) > APF (43–90%). In *P. aeruginosa*, the ethyl acetate fraction (AEF) demonstrated the highest activity with 59–69% inhibition of biofilm formation (Figure 2(a)). Much lower inhibition of biofilm formation was expressed against *E. coli* with the highest effect given by the pet-ether fraction, APF (45–67%) (Figure 2(b)).

### 3.4. Efflux Pump Inhibitory Activity of *A. macrostachya* Stem Extract and Fractions

**Figure 3:**
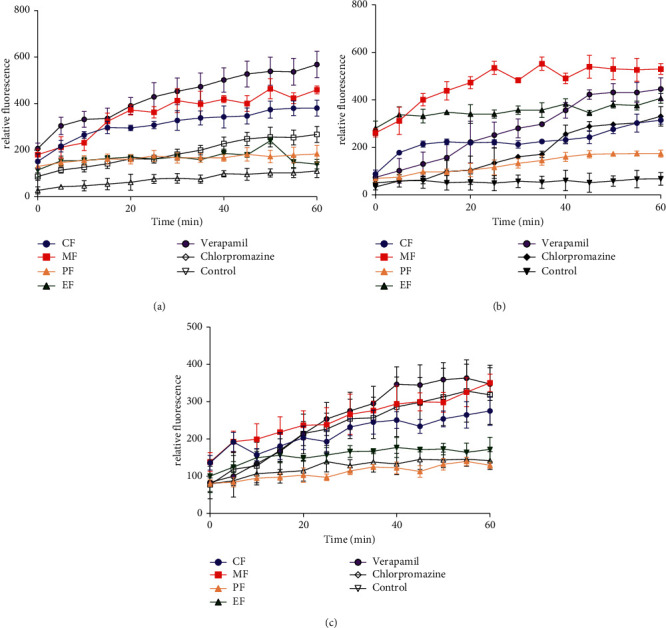
Efflux pump inhibition activity of the crude extract (CF), MeOH, EtOAc, and pet-ether fractions of *A. macrostachya* stem in *S. aureus* (a), *E. coli* (b), and *P. aeruginosa* (c).

The potential efflux pump inhibitory effect of the crude extract and fractions was assessed in the ethidium bromide (EtBr) accumulation assay.  Figure 3 displays the EtBr accumulation behaviour of *S. aureus, E. coli*, and *P. aeruginosa* in the presence of the crude extract (ACF), MeOH (AMF), EtOAc (AEF), and pet-ether (APF) fractions measured over 60 mins. This was compared to the action of two standard efflux pump inhibitors (EPIs): verapamil (VP) and chlorpromazine (CP).

In *S. aureus*, AMF and ACF (at ½ MIC) displayed remarkable efflux pump inhibition resulting in high EtBr fluorescence (Figure 3(a)). This effect was superior to that of CP but slightly lower than the effect of VP. The effects of AEF and APF were somewhat comparable to CP but relatively lower than AMF, ACF, and VP (Figure 3(a)).

In *E. coli*, both AMF and AEF (at ½ MIC) resulted in significant accumulation of EtBr with AMF having superior efflux inhibition over the standard EPIs: CP and VP (Figure 3(b)). The effect of AEF was comparable to that of VP but higher than CP. ACF showed a similar efflux inhibitory effect to that of CP. APF showed relatively much lower EPI activity (Figure 3(b)).

In *P. aeruginosa*, AMF and ACF demonstrated efflux pump inhibition activity slightly lower than VP but comparable to that of CP. The effect AEF was much lower than AMF, ACF, and the standard EPIs. APF showed little or no effect on EtBr accumulation as the fluorescence pattern was similar to the negative control (Figure 3(c)).

## 4. Discussion

This study investigated the antibacterial, antibiofilm formation and efflux pump inhibitory effects of the crude extract and three major fractions, pet-ether, EtOAc, and MeOH, from *A. macrostachya* stem bark.

In preliminary phytochemical studies, tannins, flavonoids, triterpenoids, phytosterols, coumarins, and saponins were identified in the powdered stem bark while alkaloids were absent. Results of phytochemical screening were consistent with a previous report [26]. Though alkaloids were not identified in the sample harvested from Ghana, the leafy stems collected from Burkina Faso contained some alkaloids [14]. Plant secondary metabolites such as flavonoids, tannins, phenolic acids, and triterpenoids are known to exhibit an inhibitory effect against bacteria and could contribute to the anti-infective effect of the plant seen in traditional medicine [4, 27]. High phenolic and flavonoid contents were identified in the MeOH and EtOAc fractions with the least in the pet-ether fraction.

The HT-SPOTi method is a variation of the agar dilution method used to determine the antibacterial activity of extracts against microorganisms. The crude extract and fractions demonstrated antibacterial activity at the MIC range of 250 to ≥500 *μ*g/mL *μ*g/mL. In a previous study, crude extracts and various solvent fractions from the stem bark of *A. macrostachya* demonstrated broad-spectrum antibacterial activity against selected pathogenic bacteria including *S. aureus*, *E.coli*, and *P. aeruginosa* with similar MICs ranging from 300 to 5000 *μ*g/mL [15]. Moreover, the current result was consistent with this study in that nonpolar fractions had little or no antibacterial activity while polar fractions showed a remarkable antibacterial effect. Again, the total extract (methanol) showed much higher antibacterial activity than fractionated portions suggesting a possible synergistic activity of constituents which is lost when the crude extract is fractionated [15]. In the genus *Acacia*, several species including *A. nilotica*, [28], *A. Senegal* [29], and *A. polyacantha* [30] have been reported to demonstrate potent antibacterial activity. The identified compound, stigmasterol, is also well known for its broad-spectrum antibacterial activity against several pathogenic bacteria in previous studies [18, 31].

The production of biofilms is a passive resistance strategy characterized by the formation of a polysaccharide matrix around the bacterium, which consequently leads to an obstruction of the passage of antibiotics, thus making the bacterium highly resistant to these molecules [3]. The antibiofilm-forming activity of the crude extract and solvent fractions of *A. macrostachya* stem bark was investigated by the crystal violet (CV) retention assay against three biofilm-forming bacteria: *S. aureus*, *E. coli*, and *P. aeruginosa*. This assay is based on the ability of CV, a basic dye, to nonspecifically bind to an extracellular polymeric substance secreted by bacteria for biofilm formation. The extent of binding indicates active biofilm formation which can be verified spectrophotometrically [23].

From the results, the antibiofilm-forming activity of the test samples against *S. aureus*, *P. aeruginosa*, and *E. coli*. varied according to the type of solvent fraction and was not concentration-dependent. The complexity of mixtures of active phytoconstituents distributed in the various solvents used for extraction could result in this remarkable, yet scattered antibiofilm activity observed for the test samples. This approach of investigating bioactivity based on various solvent fractions is appropriate as it can lead to the identification of specific bioactive compounds [15]. Though this is the first report of biofilm inhibitory property of *A. macrostachya*, a previous study by Tchatchedre et al. revealed that the EtOAc and EtOH extracts of *A. macrostachya* stems significantly inhibited quorum sensing (QS) in *C. violaceum* and *P. aeruginosa* [15]. Studies have shown that quorum sensing (QS) in bacteria is strongly linked to the development of biofilms. Degradation of QS signals prevents signal propagation from one region of a biofilm to another [32, 33]. The inhibition of biofilm formation observed for *A. macrostachya* in this study could be attributed to possible quenching of quorum sensing mechanisms in the bacteria. Extracts from medicinal plants have been shown to interrupt bacteria biofilm formation through mechanisms such as damaging microbial membrane structures, inhibiting peptidoglycan synthesis [34], and/or modulating quorum sensing [35]. *Acacia* species including *A. nilotica* and *A. dudgeon* have demonstrated remarkable antibiofilm-forming effects against *S. aureus*, *E. coli*, and *P. aeruginosa* [36, 37]. The marked presence of flavonoids, phytosterols, terpenoids, alkaloids, and other phenolic compounds in the genus *Acacia* [9] makes them potential candidates for the discovery of antibiofilm agents [38]. Flavonoids in particular are among the compounds that exert antibiofilm effects through the inhibition of quorum sensing [39]. Pentacyclic triterpenes and some plant sterols including stigmasterol and *β*-sitosterol have demonstrated remarkable antibiofilm activities in pathogenic bacteria [18, 40].

The intracellular accumulation of ethidium bromide (EtBr) has been used severally to confirm efflux pump inhibition using the whole-cell method [18, 41, 42]. EtBr, a known substrate of efflux pumps binds to DNA intercellularly and fluoresces when in a living organism such as bacteria. Hence, the activity of EPIs can be measured fluorometrically due to the retention of fluorescence over time. From the results, the crude extract and test fractions (except for the pet-ether fraction) of *A. macrostachya* demonstrated remarkable efflux pumps inhibition in both Gram-positive and Gram-negative bacteria comparable to standard EPIs. Some *Acacia* species have shown resistance modifying activities towards multidrug-resistant strains which produce efflux pumps. *A. Senegal* restored the antibiotic effects of chloramphenicol and florphenicol against multidrug-resistant strains of *E. coli* (strains: AG100, AG100A, AG102, AG1004 plasmid, AG100A FloR) and *K. aerogenes* (strains: Ea CM64, Ea 289, Ea 298, Ea ATCC 15038) that overproduce AcrAB or FloR pumps [43]. *A*. *polyacantha* extracts and compounds also inhibited the growth of multidrug-resistant *Staphylococcus* bacterial strains overexpressing efflux pump [30, 44].

Studies demonstrated a correlation between the QS signalling system and the control of the expression of the genes involved in active efflux systems [41]. The ability of *A. macrostachya* extracts to inhibit quorum sensing [15] could be linked to its efflux pump inhibitory effects. Efflux pumps play a significant role in the development of bacteria biofilms to result in high levels of antibiotic resistance. In effect, the inactivation of efflux pumps by EPIs could completely eradicate biofilms and reduce resistance [45]. The antibiofilm-forming and efflux pump inhibitory effect demonstrated by *A. macrostachya* in this study highlights the plant's potential as a source of bioactive compounds that could act as both EPIs and biofilm inhibitors to curb antibacterial resistance.

The current study demonstrates remarkable antibacterial activity by *A. macrostachya* stem bark. Further studies to ascertain its safety as well as identifying specific constituents responsible for the observed activities through chromatographic purification and spectrophotometric methods are considered in future works.

## 5. Conclusion

This study has provided the first evidence of the antibiofilm formation and efflux pump inhibitory potentials of the stem bark of *Acacia macrostachya*. This gives scientific credence to the traditional uses of the plant for managing infections and highlights *A. macrostachya* as a potential source of bioactive compounds to both antibiofilm and efflux pump inhibitory activity.

## Figures and Tables

**Table 1:** Phytochemical screening of the stem bark of *A. macrostachya*.

Phytoconstituent	Result
Tannin (condensed)	**+**
Reducing sugar	**+**
Phytosterol	**+**
Flavonoids	**+**
Coumarin	**+**
Saponin	**+**
Alkaloid	**−**
Triterpenoid	**+**

Note: +: detected; −: not detected.

**Table 2:** Total phenolic and flavonoid contents of *A. macrostachya* extract and fractions.

Extract/fractions	Total flavonoid content (TFC) (mean ± SD mg QCE/g)	Total phenol content (TPC) (mean ± SD mg GAE/g)
ACF	87.78 ± 2.074	44.27 ± 4.75
AMF	152.0 ± 1.234	55.43 ± 2.79
AEF	127.4 ± 16.60	28.88 ± 2.135
APF	-27.78 ± 12.36	-8.738 ± 5.725

ACF: *A. macrostachya* crude extract; AMF: *A. macrostachya* MeOH fraction; AEF: *A. macrostachya* EtOAc fraction; APF: *A. macrostachya* pet-ether fraction; QCE: quercetin equivalent; GAE: gallic acid equivalent.

**Table 3:** Minimum inhibitory concentrations of *A. macrostachya* extract and fractions.

Microorganisms	ACF	AMF	AEF	APF	Ciprofloxacin
*S. aureus*	>500	>500	>500	>500	>10
*S. pyrogens*	500	>500	500	>500	0.3125
*E. coli*	250	500	>500	>500	2.5
*P. aeruginosa*	500	500	500	>500	0.625

ACF: *A. macrostachya* crude extract; AMF: *A. macrostachya* MeOH fraction; AEF: *A. macrostachya* EtOAc fraction; APF: *A. macrostachya* pet-ether fraction.

## Data Availability

All datasets supporting the conclusions of this article are included within the article.

## Supplementary Materials

Supplementary Materials Isolation of bioactive constituents from the stem bark of *Acacia macrostachya*; spectral data for compound 1 (*β*-stigmasterol); determination of total phenolic and total flavonoid contents from the crude extract and fractions of *A. macrostachya* stem bark.
