# Supplementary material for: Antibacterial, Antibiofilm, and Efflux Pump Inhibitory Properties of the Crude Extract and Fractions from Acacia macrostachya Stem Bark
Source: ScientificWorldJournal. 2021 Oct 21;2021:5381993. doi: 10.1155/2021/5381993 (PMC8553507; doi:10.1155/2021/5381993)
Supplement: Supplementary Materials — Isolation of bioactive constituents from the stem bark of Acacia macrostachya; spectral data for compound 1 (β-stigmasterol); determination of total phenolic and total flavonoid contents from the crude extract and fractions of A. macrostachya stem bark. [file 5381993.f1.docx]

**Antibacterial, anti-biofilm and efflux pump inhibitory properties of the crude extract and fractions from *Acacia macrostachya* stem bark**

Akua Frema Barfour ^1^, Abraham Yeboah Mensah ^1^, Evelyn Asante-Kwatia ^1*^, Cynthia Amaning Danquah ^2^, Daniel Anokwah ^3^, Silas Adjei ^4^, Michael Kwesi Baah ^4^, Merlin L. K. Mensah ^4^

^1^ Department of Pharmacognosy, Faculty of Pharmacy and Pharmaceutical Sciences, College of Health Sciences, Kwame Nkrumah University of Science and Technology, Kumasi, Ghana.

^2^ Department of Pharmacology, Faculty of Pharmacy and Pharmaceutical Sciences, College of Health Sciences, Kwame Nkrumah University of Science and Technology, Kumasi, Ghana

^3^ School of Pharmacy and Pharmaceutical Sciences, University of Cape Coast, Cape Coast, Ghana

^4^ Department of Herbal Medicine, Faculty of Pharmacy and Pharmaceutical Sciences, College of Health Sciences, Kwame Nkrumah University of Science and Technology, Kumasi, Ghana

***Corresponding author**: Evelyn Asante-Kwatia

[eamireku@knust.edu.gh](mailto:eamireku@knust.edu.gh), Telephone: +233246471364

**SUPPLEMENTARY MATERIAL**

**Method**

**Isolation of bioactive constituents from *A. genipiflora* stem bark**

The ethyl acetate fraction (AEF, 18.3 g) was subjected to column chromatography (CC) using Silica gel 60 (70-230 mesh) as the stationary phase. Fractions were collected by gradient elution with pet-ether, EtOAc and MeOH in increasing order of polarity. Fifty fractions (100 ml each) were collected and bulked into 6 fractions (F1 – F6) base on TLC profiles. Further purification of fraction F3 (4.0 g) using CC eluting with pet-ether: EtOAc by gradient elution afforded 19 fractions (50 mL each) bulked into 4 sub-fractions (F3a-F3d). Fraction F3d yielded white crystals repeatedly washed with methanol to obtain compound 1 (88 mg).

**Results**

1. **Spectra data for Compound 1 (β-stigmasterol)**

**
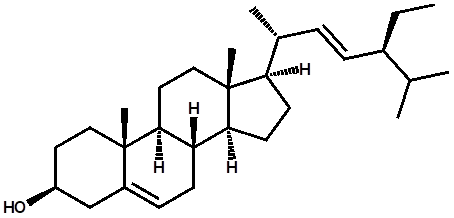
**

*Figure 1: β-Stigmasterol*

White crystals; positive EI-MS *m/z* 412.3446 [M]^+^ (calcd. for C_29_H_48_O, 412.3693). ^1^H NMR (600 MHz in CDCl_3_) and ^13^C (150 MHz in CDCl_3_) NMR data are shown in Table 1.

Table 1: ^1^H and ^13^C NMR data for Compound 1

|  | **Compound 1** | |
| --- | --- | --- |
| **Position** | **δ C** | **δ H *(J in Hz)*** |
| **1** | 36.8 (CH_2_) | 1.05, 1.81 |
| **2** | 31.2 (CH_2_) | 1.53, 1.81 |
| **3** | 71.4 (CH) | 3.50 |
| **4** | 41.9 (CH_2_) | 2.21, 2.27 |
| **5** | 140.3 (C) | - |
| **6** | 121.3 (CH) | 5.33, *d* (4.7 Hz) |
| **7** | 31.5 (CH_2_) | 1.55, 1.94 |
| **8** | 31.5 (CH) | 1.42 |
| **9** | 50.8 (CH) | 0.89 |
| **10** | 36.1 (C) | - |
| **11** | 20.8 (CH_2_) | 1.44, 1.47 |
| **12** | 40.1 (CH_2_) | 1.13, 1.99 |
| **13** | 41.9 (C) | - |
| **14** | 56.4 (CH) | 0.96 |
| **15** | 23.9 (CH_2_) | 1.03, 1.56 |
| **16** | 28.5 (CH_2_) | 1.26, 1.81 |
| **17** | 55.5 (CH) | 1.08 |
| **18** | 11.6 (CH_3_) | 0.65 s |
| **19** | 19.4 (CH_3_) | 0.98, s |
| **20** | 40.1 (CH)* | 1.32, *m* |
| **21** | 18.4 (CH_3_) | 0.90, *d* (6.8 Hz) |
| **22** | 137.9 (CH)* | 5.05, *dd* (8.4, 15.0 Hz) |
| **23** | 128.8 (CH)* | 4.85, *dd* (8.6, 15.1 Hz) |
| **24** | 50.8 (CH)* | 1.53 |
| **25** | 29.8 (CH) | 1.44 |
| **26** | 20.7 (CH_3_) | 0.94, *d* (6.3 Hz) |
| **27** | 19.8 (CH_3_) | 0.79, *d* (6.1 Hz) |
| **28** | 25.6 (CH_2_) | 1.37 |
| **29** | 11.9 (CH_3_) | 0.82, *t* (6.0 Hz) |

**2.** **Determination of total phenolic and total flavonoid contents**

***2.1 Calibration curve for determination of Total Phenolic Content***

*Figure 2: Gallic acid calibration curve for TPC determination*

***2.2 Calibration curve for determination of Total Flavonoid Content***

*Figure 3: Quercetin calibration curve for TFC determination*
